# A rare presentation of spontaneous splenic rupture from plasma cell leukaemia—a case report

**DOI:** 10.1093/jscr/rjae223

**Published:** 2024-04-11

**Authors:** Hershil Khatri, Nakhyun Kim, Tzu-Yi (Arron) Chuang, Michael Lamparelli

**Affiliations:** Department of General Surgery, Ipswich Hospital, Ipswich, QLD 4305, Australia; Department of General Surgery, Rockhampton Hospital, Rockhampton, QLD 4700, Australia; Department of General Surgery, Rockhampton Hospital, Rockhampton, QLD 4700, Australia; Department of General Surgery, Rockhampton Hospital, Rockhampton, QLD 4700, Australia

**Keywords:** case report, splenic rupture, plasma cell leukaemia, general surgery

## Abstract

Spontaneous/atraumatic splenic rupture is rare, and often associated with underlying infectious disease, or haematological malignancy. Plasma cell leukaemia (PCL) is a rare and aggressive subtype of multiple myeloma, with a higher prevalence of hepatosplenomegaly with a bleeding diathesis from secondary to thrombocytopaenia. We report the case of an 82-year-old male presenting to the emergency department with altered mentation and complaints of left abdominal pain. He presented with haemorrhagic shock. Imaging revealed a spontaneous splenic rupture. He underwent emergency laparotomy and splenectomy for which the histopathology yielded a diagnosis of PCL as the cause for rupture. He received four courses of bortezomib and hyperCVAD 1A therapy. After a long 64-day admission, he recovered well and was discharged home with outpatient haematology/oncology follow-up.

## Introduction

Multiple myeloma is a relatively uncommon haematological malignancy, affecting clonal plasma cell proliferation; plasma cell leukaemia (PCL) is a rare subset of this condition, which portends a poor prognosis with median survival of 7–14 months [1]. Patients with PCL tend to develop more severe thrombocytopaenia, a higher prevalence of hepatosplenomegaly due to extramedullary involvement, as well as renal failure when acute leukaemia is present [1]. There have only been five prior documented cases of spontaneous splenic rupture in the context of PCL, and limited understanding of the pathophysiology behind its cause [2–5]. Spontaneous (atraumatic) splenic rupture is rare and potentially life-threatening [6]. From the limited literature available, haematological malignancies and infections are the two most common causes [6].

We present a rare case of a spontaneous splenic rupture as the initial presentation of PCL in an elderly male.

## Case report

An 82-year-old male was brought to the emergency department via ambulance with acute confusion, possible seizure activity, faeculent vomiting, left-sided abdominal pain, and a 1-week history of constipation. From collateral history from family, he had been reporting atraumatic central back pain for the preceding month which had yet to be investigated.

Medical history was only significant for a nondescript seizure disorder in early childhood, essential hypertension, depression, and dyslipidaemia. He had no prior surgical history. He was a long-term heavy smoker.

The patient presented with sinus tachycardia (100 beats per minute), hypotension (systolic blood pressure 90 mmHg), and mildly hypoxaemia (O2 saturations 90% on room air). He was afebrile. On examination, he was focally tender to the left upper abdomen, but not peritonitic. He was clinically anuric.

Baseline bloods were taken (Supplementary Table 1), and fluid resuscitation was commenced. Blood tests revealed a leukaemic blood profile, thrombocytopaenia, and acute normocytic anaemia. His biochemistry was consistent with acute renal failure, hypercalcaemia, hyperphosphataemia, and hyperkalaemia.

**Figure 1:**
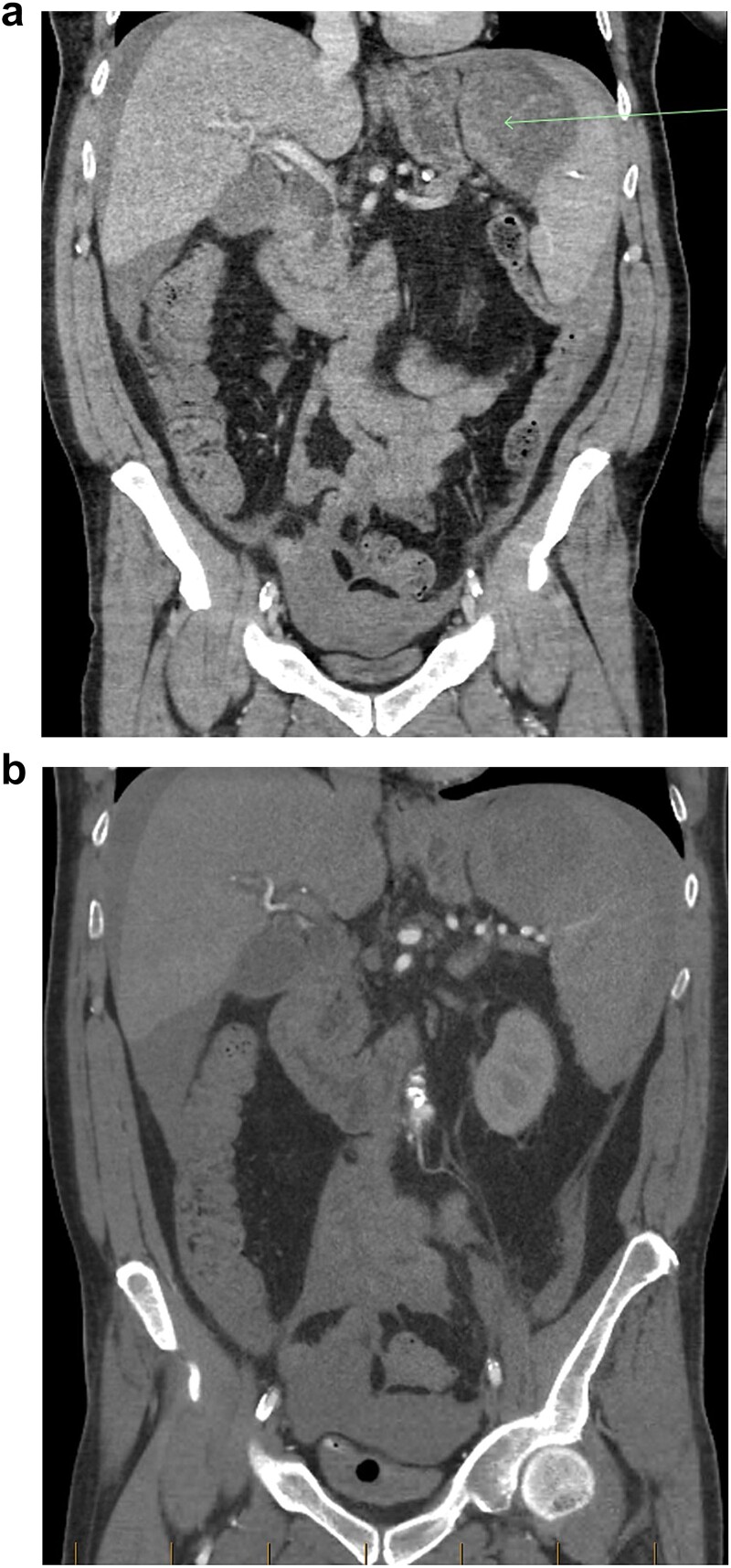
(a) Coronal view of CT-abdomen/pelvis in the portal venous phase showing moderate volume free-fluid/blood around the liver, both paracolic gutters, extending to the lower abdomen and pelvis. There is a large haematoma seen within or adjacent to the spleen (arrow). (b) Coronal view of CT-abdomen/pelvis angiogram redemonstrating the known splenic haematoma, however there was no evidence of active arterial contrast extravasation.

A baseline contrast-enhanced CT-scan of the abdomen/pelvis in the portal venous phase was organized (Fig. 1a), which demonstrated moderate intra-abdominal free-fluid/blood as well as a large splenic haematoma. There was incidental finding of a wedge-fracture of L1 with 45% loss of anterior vertebral height.

**Figure 2:**
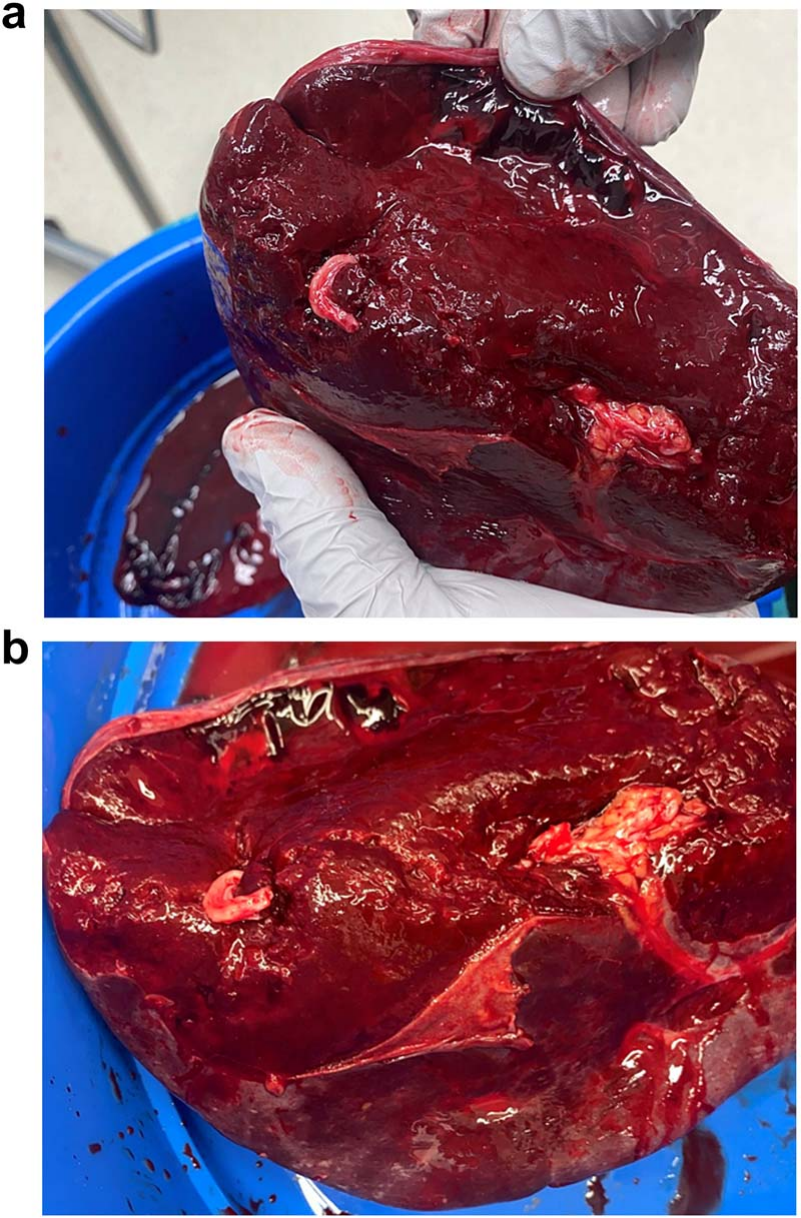
(a, b) Intraoperative specimen showing a large capsular tear with a large haematoma.

The patient responded well to intravenous (IV) fluid resuscitation. Urgent surgical and intensive care reviews were attended. It was determined that the cause for the patient’s presentation was spontaneous splenic rupture, likely secondary to occult haematological malignancy, complicated by tumour lysis syndrome, as well as anuric acute renal failure. A subsequent CT-angiogram was urgently obtained, which did not reveal and active splenic contrast extravasation. Interventional Radiology was consulted from the regional tertiary facility, whom advised that no intervention could be offered in the absence of contrast blush to indicate active bleeding. Given that the patient was responsive to fluid resuscitation, multi-disciplinary decision was that the patient should be medically optimized with dialysis before pursuing surgical intervention, and he was subsequently admitted to intensive care for monitoring. Within 3 hours of admission however, the patient became progressively shocked, with an acute drop in haemoglobin (88 to 61 g/L) as well as platelet count (90 to 63 × 10^9 /L). An emergency laparotomy/splenectomy was performed overnight. Intra-operatively there was large volume free blood and clots within the abdominal cavity. There was a large capsular tear of the superior pole of the spleen (Fig. 2a and b), and total splenectomy was performed. Total estimated blood loss was 4.5 L. He received a total of 5 L of crystalloid, 8 units of packed red blood cells, 1 g tranexamic acid, 1 unit of platelets, 6 units of cryoprecipitate, 2 units of fresh frozen plasma, as well as 1.3 L of cell-saver blood.

The patient was stabilized post-operatively, and subsequently transferred to a tertiary hospital intensive care unit, under the care of haematology for chemotherapy. Further bloodwork showed a high kappa-free light-chains of 10 000, with circulating plasma cells on peripheral smear. His histology of spleen demonstrated splenic involvement by plasma cell myeloma/PCL. He received four courses of bortezomib and hyperCVAD 1A therapy. After a long 64-day admission, he recovered well and was discharged home. Unfortunately, the patient passed away 1 year later from an unrelated cause.

## Discussion

Splenic rupture in the absence of trauma is uncommon, however clinicians must maintain a high index of clinical suspicion in patients presenting with left upper abdominal pain and haemodynamic instability [6]. CT and ultrasound are increasingly available imaging tools to aid clinicians in determining the extent of damage, as well as help guide management options (conservative vs surgical). Splenic injuries are commonly graded using the American Association for the Surgery of Trauma (AAST) scale. Grades I–III injuries are considered mild–moderate injuries and can generally be managed non-operatively; grades IV–V are high-grade injuries with high mortality risk and generally necessitate surgical intervention [7]. Non-operative management has previously been trialled for patients with high-grade injuries that are haemodynamically stable, and there are no signs of active bleeding [8, 9]. A trial of conservative medical optimization was offered for this patient with a grade IV injury, however unfortunately they deteriorated shortly thereafter which ultimately necessitated emergent laparotomy/splenectomy.

### Learning Points

1) Spontaneous splenic rupture is a rare and potentially fatal complication of haematological malignancies.
2) PCL is a rare and aggressive disease, with unique pathophysiology predisposing to spontaneous splenic rupture more than other haematological conditions.
3) Surgeons/surgical trainees should have a high index of suspicion in patients presenting with shock and isolated left abdominal pain.
4) Conservative management is unlikely to be successful in high-grade splenic injuries (grades IV–V), and surgeons should have a low-threshold to proceed with surgery in unstable patients.

## Supplementary Material

APPENDICES_rjae223

## Data Availability

Data sharing is not applicable to this article as no new data were created or analyzed in this study.
