# Supplementary material for: A rare presentation of spontaneous splenic rupture from plasma cell leukaemia—a case report
Source: J Surg Case Rep. 2024 Apr 11;2024(4):rjae223. doi: 10.1093/jscr/rjae223 (PMC11007546; doi:10.1093/jscr/rjae223)
Supplement: APPENDICES_rjae223 [file appendices_rjae223.docx]

**APPENDICES**

Appendix A: Patient’s bloods at time of presentation to hospital

Table 1: Patient's blood tests at time of presentation

| Test | Value | Units | Reference Range |
| --- | --- | --- | --- |
| Haemoglobin (Hb) | 88 | g/L | 135 – 180 |
| Haematocrit (Hct) | 0.27 |  | 0.39 – 0.52 |
| Platelet Count (Plt) | 90 | x 10^9 /L | 140 – 400 |
| White Cell Count (WCC) | 48.8 | x 10^9 /L | 4.0 – 11.0 |
| Neutrophils | 13.26 | x 10^9 /L | 2.00 – 8.00 |
| Lymphocytes | 11.36 | x 10^9 /L | 1.00 – 4.00 |
| Film Comment | Other cells (46%) are pleomorphic with basophilic cytoplasm including immature large cells with high N:C ratio and prominent nucleoli, intermediate more mature cells with multiple inconspicuous nucleoli, reactive forms with clefting and lymphoplasmacytoid forms. Concerning for high grade lymphoma with other differential acute leukaemia, correlate with flow cytometry | | |
|  | | | |
| Creatinine | 1230 | μmol/L | 60 – 110 |
| Estimated Glomerular Filtration Rate (eGFR) | 4 |  | >90 |
| Lactate Dehydrogenase (LDH) | 1749 | U/L | 120 – 250 |
| Corrected Calcium | 3.59 | mmol/L | 2.10 – 2.50 |
| Phosphate | 3.42 | mmol/L | 0.75 – 1.50 |
| Potassium | 5.8 | mmol/L | 3.5 – 5.0 |
|  | | | |
| K/L Ratio (N Latex) | 1100 |  | 0.31 – 1.56 |
| Lambda Free Light Chains (N Lambda) | 10 | mg/L | 8 – 27 |
| Kappa Free Light Chains (N Lambda) | 10000 | mg/L | 7 – 22 |

Appendix B: Histopathology / flow-cytometry results

| Test | Report |
| --- | --- |
| Histopathology (Spleen) | MACROSCOPIC  An intact spleen measuring 170x120x60mm with a weight of 456 grams. Over the hilar surface the capsular surface is disrupted and this is associated with adherent blood clot. No obvious laceration is seen. No hilar lymph nodes identified. The cut surface of the spleen shows no focal lesions.  MICROSCOPIC:  Sections of the spleen show white pulp atrophy along with expanded red pump by a lymphoplasmacytic infiltrate. Mitotic figures are readily identified. Haemosiderin laden macrophages are also present. The plasma cells are positive for CD138 and MUM1 and show kappa light chain restriction. The cells are negative for CD20, CD79a and CD56. Ki 67 shows a proliferation index of 70%. There is a mixed population of T and B cells and histiocytes in the background. EBER shows non-specific staining.  Flow cytometry on bone marrow shows aberrant plasma cells.  Bone marrow shows features of plasma cell myeloma manifesting as plasma cell leukaemia.  The overall features would be in keeping with splenic involvement by plasma cell myeloma/plasma cell leukaemia. |
|  | |
| Flow cytometry (peripheral blood) | IMMUNOPHENOTYPE Positive: CD38, CD56 (weak), and CD138  Negative: CD19, CD20, CD34, CD45, CD117 and HLA-DR  COMMENT  Approximately 50% of the total cells were identified in the cd45 weak to negative region and demonstrate the above immunophenotype. This population is consistent with aberrant plasma cells.  These cells were negative for B-cell markers and surface light chains.  An abnormal clonal B-cell population was not identified.  Approximately 0.5% of the total cells expressed CD13, CD33, CD34, CD117 and HLA-DR, consistent with normal myeloblasts.  There was no evidence of a significant population of cells that expressed CD10, CD19 and CD34, consistent with normal precursor B-cells/  The T-cell immunophenotype was unremarkable. |

Appendix C: Radiological Reports

CT-ABDOMEN & PELVIS WITH CONTRAST (PORTAL VENOUS PHASE):

*Findings:*

There is haemorrhage noted in the upper abdomen around the spleen and anterior to the stomach. There is suboptimal enhancement and heterogeneity with artefact degrading the images of the upper abdomen. No large tear of the spleen identified however a small tear cannot be confidently excluded in this study. Artifact also degrading the images of the liver. No focal lesion of the liver identified. The gallbladder, pancreas, adrenal glands, and kidneys are unremarkable. There is no perinephric haematoma or collection. There is no hydronephrosis.

There is fluid/haemorrhage noted around the liver and both paracolic gutters extending to the lower abdomen and pelvis.

Small atelectasis of right lung base is noted. No pleural effusion identified.

Wedging fracture of superior endplate of L1 with approximately 45 percent loss of anterior vertebral height is noted.

*Conclusion:*

There is haemorrhage noted in the upper abdomen extending into the paracolic gutters to the lower abdomen and pelvis. There is large haematoma seen adjacent to the spleen. There is artifact degrading the images of the abdomen and splenic tear cannot be excluded.

Urgent surgical review is recommended.

CT-ABDOMEN ANGIOGRAM:

*Findings:*

Good enhancement of the splenic arteries noted. There is no evidence of extravasation of contrast or leak to suggest active bleeding. Two calcific foci seen in the spleen suggestive of old granuloma. There is no blush of contrast seen in the abdomen. Haematoma noted around the spleen and fluid/haemorrhage noted in the upper and lower abdomen and pelvis.

*Conclusion:*

No extravasation of contrast or active bleeding identified.
